# Influence of Choline Chloride/Urea and Glycerol Plasticizers on the Mechanical Properties of Thermoplastic Starch Plastics

**DOI:** 10.3390/polym16060751

**Published:** 2024-03-09

**Authors:** Jacob Staker, Sydney Schott, Riya Singh, Kourtney Collier, Gregory Druschel, Amanda P. Siegel, Andres Tovar

**Affiliations:** 1 Department of Chemistry and Chemical Biology, Indiana University-Purdue University Indianapolis, Indianapolis, IN 46202, USA; jsstaker@purdue.edu (J.S.); apsiegel@iu.edu (A.P.S.); 2 Department of Mechanical and Energy Engineering, Indiana University-Purdue University Indianapolis, Indianapolis, IN 46202, USA; 3 Department of Earth and Environmental Sciences, Indiana University-Purdue University Indianapolis, Indianapolis, IN 46202, USA

**Keywords:** compostable plastic, deep eutectic solvent, film characterization, chemical structure

## Abstract

Bio-based plastics made of food-safe compostable materials, such as thermoplastic starch (TPS), can be designed into films that have potential to replace many non-biodegradable single-use plastic (SUP) items. TPS film characteristics, such as elongation at break and tensile strength, are largely affected by the choice of the plasticizers used in formulation. Our work identifies the mechanical properties and the chemical structural differences between TPS films made with two different plasticizer mixtures that have not yet been compared alongside one another: deep eutectic solvent choline chloride/urea (1:2) (CC:U) and glycerol with an acetic acid catalyst (AA:G). Potato-based TPS samples were formed by mixing each plasticizer with a consistent amount of potato starch and distilled water with heat. After gelation formation, the viscous TPS mixture was centrifuged to degas and extruded. Films were dried at controlled room temperature. Characterization included the tensile testing of coupons according to ASTM (American Society of Testing and Materials) standard D638, attenuated total reflectance Fourier-transform infrared (ATR-FTIR) spectroscopy, X-ray diffraction (XRD), melting point (MP), and scanning electron microscopy (SEM). The AA:G films displayed significantly higher tensile strength (M = 2.04 ± 1.24 MPa) than the CC:U films (M = 0.18 ± 0.08 MPa); however, the CC:U films had higher elongation at break (M = 47.2 ± 3.6%) than the AA:G films (M = 31.1 ± 12.6%). This can be explained by the difference in functional groups, composition, and the degree of crystallinity evidenced by the FTIR, XRD, MP, and SEM results. Our findings suggest that potato-based TPS films with an AA:G plasticizer mixture hold promise for SUP applications that require more strength, while CC:U films may be more suited for wraps and bags that require flexibility. These innovations can aid to mitigate the environmental impact of harmful plastic waste.

## 1. Introduction

Bio-based plastics, produced from food-safe thermoplastic starch (TPS), can potentially replace non-biodegradable single-use plastics (SUPs), especially those commonly used in food consumption, packaging, and storage. For decades, fossil-based plastics such as polystyrene (Styrofoam) and polyethylene (ubiquitous in everyday plastic bags) and their derivatives have contributed to the accumulation of unwanted SUPs in natural environments [1]. This pressing issue is exemplified by the shifting island of plastics in the Pacific Ocean and degraded microplastics in diverse ecosystems [2]. Motivated by an escalating awareness of the detrimental effects of non-biodegradable plastics, there is a clear trajectory in favor of bio-based plastic alternatives. This shift is evidenced by the anticipated global market growth of the bio-based plastic market size from USD 11.5B in 2022 to USD 27.3B by 2027 [3].

In the past two decades, there has been a surge in research and development efforts to incorporate TPS into bio-based plastics. Efforts have been directed towards integrating starch and agro-waste into synthetic polymers such as polyvinyl alcohol (PVA) [4] and polylactic acid (PLA) [5,6]. Although such polymers containing starch and other organic components may undergo degradation, there is also the potential for generating microplastics that could leach into the environment. To address this concern, newer variations of 100% organic and compostable TPS plastics have been recently explored. Starch derived from various plants and staple foods, such as cassava (tapioca) [7], corn [8], sugar palm [9], rice [10,11], and potato [12,13,14], has demonstrated effectiveness in different TPS formulations [15]. Incorporating natural fibers such as bamboo and sugarcane has been shown to enhance the mechanical properties of TPS films, all while preserving compostability [16]. Currently, TPS films hold promise for applications in agriculture, as plastic mulch [17], and in medicine, as seen in the production of disposable pill capsules [18]. The potential for a wider range of applications for TPS films, including their use as substitutes for widely consumed SUP items and plastic bags, is contingent upon the ability to provide appropriate mechanical properties to these films.

A critical component that determines the TPS film’s mechanical properties (tensile strength and elongation at break) is the plasticizer. The choice of plasticizer has the potential to change intermolecular and intramolecular bonds in the starch backbone, which can have profound effects on material properties. Two types of plasticizers commonly employed in TPS films are polyols and ionic liquids. A typical polyol is glycerol [19,20] and can be paired with acetic acid, which will serve as a “cleaver” for certain glycosidic bonds in carbohydrates and act as an acid catalyst [12]. Common ionic liquids include deep eutectic solvents (DESs), such as choline chloride and urea [21,22,23,24,25]. However, a side-by-side comparison between the glycerol (AA:G mixture) and choline chloride/urea (CC:U) plasticizers is yet to be conducted.

In this work, we compare the effect of AA:G and CC:U plasticizer mixtures on the mechanical properties of TPS films made with potato starch. We incorporate a consistent amount of deionized water solvent and potato-starch polymer backbone to identify chemical, structural, and material characteristic distinctions. Our methods involve the tensile testing of plastic films according to the ASTM Standard D638 [26], and the analysis of chemical structures through Fourier-transform infrared (FTIR) spectroscopy with attenuated total reflectance (ATR), scanning electron microscopy (SEM), X-ray diffraction (XRD), and melting point (MP) determination. The analysis of chemical structures provides insight into the properties of TPS and their potential to replace SUPs derived from fossil fuels. This possibility is substantiated by the presence of hydrolysable polysaccharide polymer chains, which can function as substitutes for hydrocarbon chains traditionally produced through non-green processes.

The main mechanism underlying TPS formation is gelatinization, which occurs from heating, and is often aided by partial hydrolyzation [27]. Notably, potato starch is a heterogeneous mixture of amylose and amylopectin polymers, each composed of glucose monomers linked by glycosidic α(1 → 4) bonds, with amylopectin being slightly branched and containing some α(1 → 6) bonds. The content variation of these polymers is attributed to the nature of the glycosylic bonds present in each polymer chain macrostructure. When heated in the presence of water, starch undergoes gelatinization, an endothermic transition which enables starch granules to absorb water and swell. Incorporating a plasticizer enables TPS to maintain a swollen, and in most cases, a more flexible structure even after solvent evaporation. The addition of acetic acid and heat causes some hydrolysis of glycosidic bonds, the extent of which will be analyzed below. As a DES, incorporating choline chloride and urea into TPS also affects the polymeric structure.

**Figure 1:**
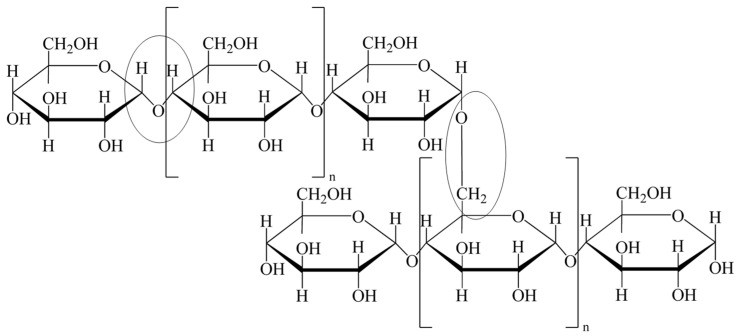
TPS main polymer backbone of amylopectin’s molecular structure showing both α(1 → 4) (left oval) and α(1 → 6) (right oval) glycosidic linkages. Amylose contains only α(1 → 4) linkages.

Compounds with polar groups in a terminal position, such as glycerol, have the ability to act as a crosslinker due to their affinity to hydrogen bond, pulling other polar groups on a molecule or polymer chain intramolecularly together. Similarly, other polar compounds such as urea also have the ability to create such bonds—similar to the usage of polyvinyl alcohol plastics with varying inorganic salts [28]. This presents a wide variation of potential applications. The hydrolyzation process can in theory take place at either glycosidic linkage, α(1 → 4) (left oval) or α(1 → 6) (right oval) (Figure 1), but in practice, the α(1 → 4) is stronger and less likely to be cleaved. The hydrolyzed chains have the potential to reform linkages. Plasticizers and crosslinkers are then utilized as they conjugate these new polymer chains together into a colloidal macrostructure in solution. The gel will sit in this state until a dehydration and evaporation process occur allowing the removal of the solvent and the acid catalyst.

Changes in the plasticizer’s composition and abundance can shift characteristics such as tensile strength and elongation at break. ATR-FTIR spectroscopy can determine chemical functionality differences when changing plasticizers. SEM and XRD can suggest changes in crystallinity within the TPS structure. MP determination provides insight into TPS’s ability to withstand higher temperatures with different plasticizers. Utilizing such instrumentation allows us to characterize the material differences between TPS formed from the two plasticizers, enabling us to understand how they can be used with starch polymers for different applications and better realize the full potential of compostable plastics.

## 2. Materials and Methods

### 2.1. Materials

The materials used in this study were sourced as follows: potato starch was obtained from Gefen Foods (Bayonne, NJ, USA); distilled white vinegar (5% acetic acid) from Walmart’s Great Value (Bentonville, AR, USA); choline chloride from PR1MA (Fenton, MO, USA); urea from Thermo Scientific (Fair Lawn, NJ, USA); deionized (DI) water from Sigma-Aldrich (Burlington, MA, USA); and vegetable glycerol from Florida Laboratories (Fort Lauderdale, FL, USA).

All ingredients utilized in this study adhere to the Food Chemical Codex (FCC) grade standards, ensuring their compostability. The TPS formation process outlined in Section 2.2 is designed to preserve the compostability characteristics of these ingredients and the TPS films.

### 2.2. Preparation of Choline Chloride/Urea and Acetic Acid/Glycerol TPS Films

The CC:U mixture (in a 1:2 ratio) was synthesized by adding a mole/mole amount of choline chloride and urea into a 5 mL vial to mix with heat until fully melted and homogenous. A clear solution was left that stayed liquid at room temperature. The formation of the CC:U potato-based TPS started by transferring the CC:U mixture with 100 g of DI water and 5 g of potato starch. This mixture was then stirred with heat up to 60 °C, where a viscosity change occurs, showing gelatinization.

The formation of plastic samples from AA:G (in a 0.04:1 ratio) was accomplished by mixing 1.5 g of distilled white vinegar (AA source) with 2 g of vegetable glycerol and mixing in 100 g of DI water until fully combined. This was used to dissolve 5 g of potato starch, followed by heating up to 70 °C where, similarly, the mixture becomes more viscous, demonstrating gelatinization.

**Figure 2:**
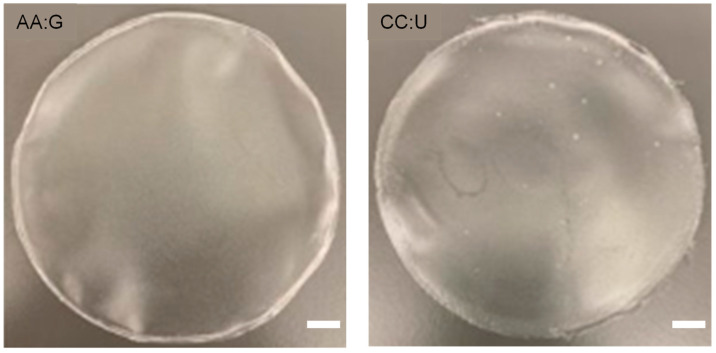
TPS films extracted from the Petri dish after drying for four days. Slight visible differences are found between the AA:G film (**left**) and the CC:U film (**right**). Scale bar = 10 mm.

Each viscous TPS mixture was extruded to 50 mL centrifuge vials and centrifuged at 2000 RPMs for 2 min to degas. For all film samples, about 14 g of gel was poured into a Petri dish and was left to sit in controlled laboratory ventilated air at room temperature (20–23 °C) to dehydrate naturally for four days before testing (Figure 2). Evaporation was monitored by weighing the samples at the time of extrusion and when fully dried, resulting in about 1.3 g of plastic with 0.16 to 0.20 mm thickness for all film samples. Film thickness for the purposes of determining tensile strength was measured with a digital micrometer (0–25 mm, ±0.001 mm). Three measurements were taken for each film sample, and then averaged.

### 2.3. ATR-FTIR Spectroscopy

Fourier-transform infrared (FTIR) spectroscopy with attenuated total reflectance (ATR) was used to analyze the presence of functional groups. This nondestructive test was performed on fully dried TPS film samples utilizing a Nicolet iS5 equipped with a single-bounce diamond crystal from Thermo Fisher Scientific (Waltham, MA, USA).

### 2.4. SEM Microscopy

SEM microscopy was performed by taking a small coupon from the TPS films and analyzing it using a JSM-7800F Field Emission Scanning Electron Microscope (JEOL, Ashland, OR, USA) with 5.0 kV at ×330 magnification. Samples were cut with a metal blade cutter. The cut films were placed at the desired angle by using adhesive tape to adhere them to the side of the SEM stage.

### 2.5. Tensile Testing

TPS film samples were prepared to fit coupon-sized rectangles according to ASTM standard D638. Tensile strength and elongation at break were determined via tensile testing on a universal testing machine (UTM) from Jinan Focus Test Instrument Co., Ltd. (Jinan, China).

### 2.6. X-ray Diffraction Testing

After drying, the TPS films were cut into strips before being set on a glass slide for XRD analysis using a Bruker D8 Discover instrument (Billerica, MA, USA). Additionally, native potato starch powder was put onto a separate glass slide for comparative analysis. The output data including intensity (arb. units) and diffraction angle (2θ) were exported and normalized before plotting (Section 3.3).

### 2.7. Melting Point Determination Using the Capillary Method

A small quantity (~0.2 g) of pure potato starch and each TPS film was put into a mortar and pestle and ground until a powder. Capillary tubes were used for loading the powders into an SRS DigiMelt MPA160 (Sunnyvale, CA, USA). The first sign of melting was recorded at that temperature as a lower bound. The upper bound was recorded when the solid was completely melted or burnt. All experiments started at 60 °C, and the heat ramp was 10 °C/min until the sample was fully dissolved or burnt.

## 3. Results

### 3.1. Choline Chloride/Urea and Glycerol Infrared Spectroscopy

**Figure 3:**
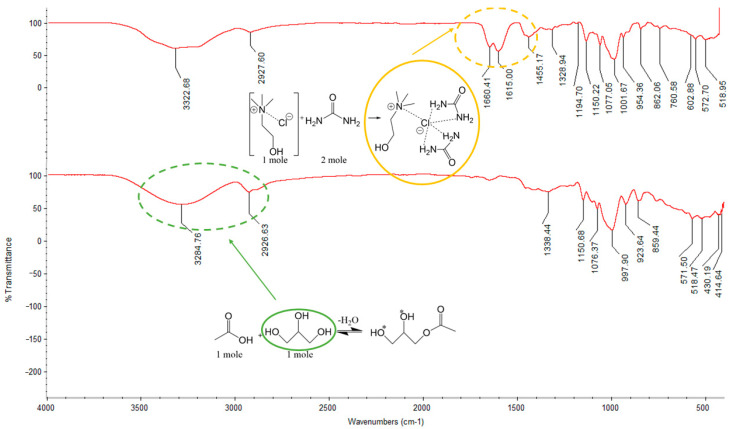
Choline chloride/urea (CC:U) deep eutectic solvent (**top**) and acetic acid/glycerol (AA:G) mechanism (**bottom**) with ATR-FTIR spectroscopy. Significant differences in spectra are shown with dashed ovals (yellow for CC:U plastic and green for AA:G plastic) on the spectra and the chemical components they correspond to are shown with solid ovals.

Within plasticizer-enhanced TPS films, changes in the functional groups present in the plasticizing site significantly alter the plastics’ overall characteristics. FTIR spectroscopy allowed the determination of the functionality of the plastics made in our lab. Figure 3 (top) shows the presence of CC:U DES incorporated into the film. The presence of hydrogen bonding enables DES to act as a plasticizer. The significant functional groups acting as plasticizer include the urea (stretches at 1660, 1615, and 1455 cm^−1^) and a hydroxyl groups. Another peak present only in the CC:U system is the C-N stretch (1455 cm^−1^). In relation to the FTIR spectroscopy data gathered, the presence of these functional groups still appears after the drying phase.

In Figure 3 (bottom), the broad peak centered at 3284 cm^−1^ indicates the strong presence of the hydroxyl functional group (-OH) which can be attributed to excess water, potato starch, and or polyol (glycerol) in the AA:G plasticizer. The peak is less broad in the CC:U as there is no glycerol plasticizer added. Additionally, the absence of carbonyl stretches, which would be expected if any acetin derivatives were made or acetic acid remained in the film, suggests that acetic acid has evaporated. Interestingly, the stretches between 950 and 1150 cm^−1^ are very similar in both films, and FTIR is unable to suggest whether any rearrangement of amylose and amylopectin chains has occurred.

FTIR spectroscopy of CC:U plastic: Vmax/cm^−1^ 3323 (NH), 2928 (CH), 1660 (CO), 1615 (NH), 1455 (CN), 1329 (CO), 1195, 1150, 1077, 1002, 954 (COH), 862–519. ATR-FTIR spectroscopy of AA:G plastic: Vmax/cm^−1^ 3284 (OH), 2927 (CH), 1338 (CO), 1151, 1076, 998 (COH), 923–415 (*Hydroxyl groups could also be deprotonated and turned into ester groups, allowing di and triacetin products in the presence of enough acetic acid).

### 3.2. Scanning Electron Microscopy Images

**Figure 4:**
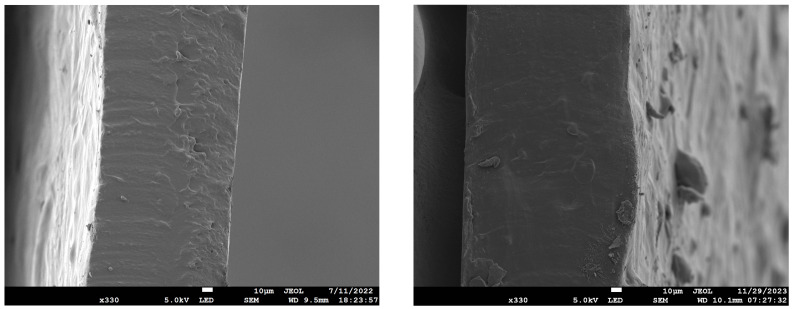
Scanning electron microscopy of AA:G (**left**) and CC:U (**right**) TPS films. Scale bar = 10 μm (×330 magnification).

Figure 4 shows cross-sectional images of the AA:G (left) and CC:U (right) TPS films. Both appear reasonably homogeneous, although a very few blobs, which might be individual potato starch granules, can be seen in the film cross-section and on the surface of the CC:U film. While both films are very homogeneous, the AA:G film appears more brittle due to its fracture surface in comparison with the CC:U film, which appears more amorphous/plastic.

### 3.3. X-ray Diffraction Data

**Figure 5:**
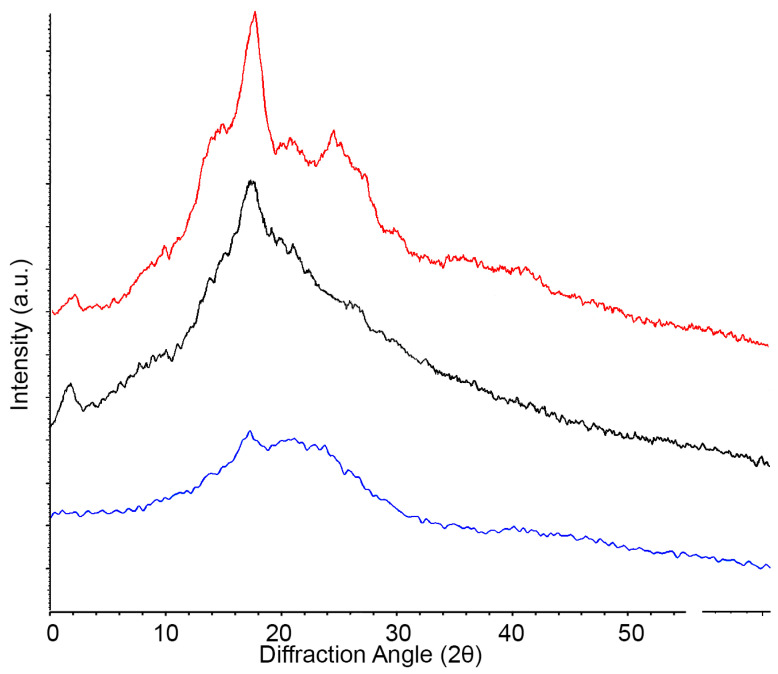
X-ray diffraction (XRD) of native potato starch (red), AA:G TPS films (black), and CC:U TPS films (blue).

XRD data from native starch (red), glycerol (AA:G) films (black), and CC:U films (blue) are shown in Figure 5. The native starch yielded sharp peaks at 2, 17, 22, and 24 2θ(°), with other pronounced peaks at 15 2θ(°), demonstrating a relatively high degree of crystallinity, common for an organic starch powder. Similar peaks have been reported elsewhere [29]. The AA:G films also showed reasonably high crystallinity and retained the peaks at 17, and a shoulder at 22 2θ(°), but demonstrated a broadening of peaks at 1–2.5 and 10–14 indicative of the gelatinization and modification of the internal structure.

The CC:U films had even broader peaks than either native starch or the AA:G films (peaks at 17 and 22 2θ(°)), demonstrating far less internal structure than either the native starch or the AA:G films and a nearly total loss of crystallinity. This result is similar to a TPS created using potato starch and CC:U prepared using a different method [25]. The XRD of neat amylose displays peaks at 2–5.5, 17, and 20, whereas the XRD of neat amylopectin displays peaks at 15, 17, and 23 [25]. The reduction in all of the peaks, and particularly at 17 and 24, suggests that CC:U causes a significant degradation of glycosidic bonds in amylopectin and amylose. Certainly, the results show that CC:U had a larger impact on crystallinity than AA:G. As we would expect, that increased crystallinity would lead to increased mechanical strength, these results suggest that CC:U films may be mechanically weaker than AA:G films, but more flexible.

### 3.4. Tensile Strength and Elongation at Break Percentage Data

**Figure 6:**
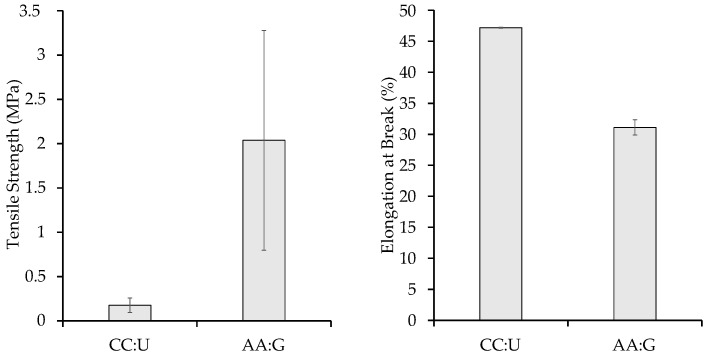
Tensile strength and elongation at break of CC:U and AA:G TPS films.

The tensile strength of the CC:U film averaged 0.176 MPa (SD = 0.080) while that of the AA:G film averaged 2.037 MPa (SD = 1.240). The elongation at break of the CC:U film averaged 47.16% (SD = 3.57), while that of the AA:G film averaged 31.08% (SD = 12.59). These results and the film sample thickness are summarized in graph form in Table 1 and illustrated in Figure 6.

### 3.5. Melting Point Determination

Further characterization of the films compared melting point ranges. A pure potato starch sample degraded at 260 °C. The starch turned brown and burned; however, it did not melt due to its mainly amorphous structure. The melting point range of the TPS film with the AA:G plasticizer was observed to be 195–212 °C. It should be noted that the sample started to brown and “sweat” at the lower bound. The melting point range of the TPS film with the CC:U plasticizer was observed at 226–240 °C. This sample also started to brown and “sweat” as temperature increased.

## 4. Discussion

Significant functional groups shown in Figure 3 (top) for the plasticizer site produce specific characteristics. The CC:U TPS showed the three peaks present for the urea compound at 1660 cm^−1^, 1615 cm^−1^, and 1455 cm^−1^. Utilizing materials containing polyurethane show similar N-H and C=O peaks around 1650 cm^−1^ and 1530 cm^−1^, respectively [30]. In contrast, the AA:G TPS undergoes different chemistry than the CC:U TPS due to the mixture’s presence of acetic acid. With only a small amount of acetic acid present, few amounts of the mono-, di-, or triacetin product are formed. However, there is glycosidic bond cleavage and hydrolysis. Any remaining acetic acid likely evaporates during the drying stage, as it is not seen by FTIR. There was no evidence of significant ester (CO) peaks which would have been reported in ~1700 cm^−1^. However, present were a OH broad peak (3284 cm^−1^), a peak at 998 cm^−1^ (COH), and the CH peak at 2927 cm^−1^ similar to the CC:U CH peak.

While the ATR-FTIR results depict how the chemical functionalities differ based on the chosen plasticizer, the X-ray diffraction data shed further light on differences between the plasticizers. From Figure 5, we see that the native potato starch itself has a structure with three distinct peaks at 2, 17, and 22 2θ (°). We also see that the AA:G TPS films yielded an increase in structure, as the peaks are in higher intensities, relatively speaking, at those same angles. The CC:U TPS film XRD data showed a significant loss of structure, where only a few of the peaks from potato starch remain. The XRD data identify significant loss of crystallinity in the CC:U sample, which appears consistent with the material characteristics and explains why CC:U’s elongation at break is much larger than AA:G’s. In Figure 2 and Figure 4, it is observed that the CC:U film exhibits a homogeneous gel-like surface layer compared to the homogeneous compact surface layer in the AA:G film. This is consistent with the difference in crystallinity.

For thermal analysis, as determined by the capillary method, pure starch experimentally degraded fully at 260 °C. This value is consistent with the results reported by Thomas Scientific (thomassci.com accessed on 1 March 2024), which indicate a TPS melting point (MP) in the range of 255 to 258 °C. As expected, adding a plasticizer was observed to lower the MP of the TPS films. The MP for the CC:U film ranged from 226 to 240 °C, while the AA:G film had a lower MP in the 195 to 212 °C range. Other studies have reported an MP for TPS with glycerol in the ranges of 180 to 188 °C [13] and 150 to 200 °C [31], which are consistent with the findings of this work.

Notably, the composites’ melting points typically fall between their constituents’ melting points. Glycerol, the plasticizing compound in the AA:G film, has a relatively low melting point (MP) ranging from 17 to 20 °C, whereas CC:U, being a deep eutectic solvent, has an even lower MP [22,25]. Surprisingly, our observed MP values differ from the expected trend, as the AA:G films exhibit a lower MP than the CC:U films. This discrepancy may be attributed to the limitations of the capillary method employed. Differential Scanning Calorimetry (DSC) is a more suitable method for determining melting points, especially for amorphous polymers. Hence, we plan to utilize DSC in future studies to obtain more accurate results. Obtaining precise MP values is important, particularly when producing TPS single-use films for high-temperature applications.

Both CC:U and AA:G samples were tested via ASTM standard D638, a standard used by others [12,19], resulting in tensile strength differences between the plastics after drying (Table 1). Tensile strength and elongation at break have also been tested on TPS made from corn starch where strength results with glycerol were as high as 2.242 MPa [8], though they did not utilize an acid catalyst and had larger samples with more material. Tensile strength with corn stover-derived cellulose films has also been shown to be as high as 25 MPa; however, this was achieved with incorporating PVA, making it less compostable [27]. Indeed, there is still room to improve our 100% biodegradable and compostable formulations for increased strength. Internally, compared to the CC:U films, the AA:G films demonstrated around four times greater tensile strength. The glycerol crosslinker creates more uniformity and sturdiness within the plastic due to glycerol’s more constricted utilization of H-bonding. If any acetin derivatives were present, these too would allow H-bonding with its increased number of OH sites. In comparison to the AA:G films, the CC:U films had greater elongation at break. As expected, the addition of the DES, creating a plasticizing complex where ionic intramolecular interactions are taking place in the plasticizer, increased the elongation property for the macrostructure interactions with the polymer backbone, which explains the increased flexibility.

In contrast to the commercial fossil-based low-density polyethylene (LDPE) film commonly utilized in single-use plastic grocery bags, the mechanical properties of CC:U and AA:G TPS films are notably lower in regard to tensile strength and elongation at break. The LDPE film’s tensile strength at yield (machine direction) falls within the range of 10 to 15.2 MPa (matweb.com accessed on 1 March 2024), whereas for the AA:G TPS film, the ultimate tensile strength averages 2 MPa. Similarly, the elongation at break for the LDPE film (machine direction) ranges from 150 to 580% (matweb.com accessed on 1 March 2024), while for the CC:U TPS film, it averages 48%. However, in comparison with melting points, our CC:U TPS contained resistance up to 226 °C and the AA:G TPS up to 195 °C, whereas LDPE has a melting point of 105–115 °C (Omnexus.com accessed on 1 March 2024). This improved melting point provides more room for applications that could potentially be exposed to higher temperatures.

## 5. Conclusions

Our study compares the mechanical properties and chemical structural differences of TPS films using two different plasticizers, choline chloride/urea (1:2) (CC:U) and acetic acid/glycerol (0.04:1) (AA:G), generated by mixture with water and gentle heating. The AA:G films exhibited significantly higher tensile strength compared to the CC:U films, while the CC:U films demonstrated higher elongation at break than the AA:G films. These distinctions can be attributed to variations in functional groups, composition, and crystallinity observed in the FTIR, XRD, and SEM results.

The rigid and robust nature of TPS films produced with the AA:G plasticizer mixture suggests that AA:G compostable plastics have the potential to serve as viable replacements for non-biodegradable single-use plastics (SUPs) in consumer packaging. This recommendation is supported by both the cost-effectiveness and the complete compostability of all ingredients within this formulation. Similarly, TPS films incorporating the CC:U plasticizer exhibit characteristics that make them particularly suitable for SUP bags and wraps. There is still considerable potential for improvement in the development of compostable films, as indicated by the comparison of the inherent strengths of less compostable and non-biodegradable formulations.

One limitation of this study is the lack of testing on various concentrations of plasticizer or investigation into different processing parameters, such as gelatinization time and temperature. These unexplored factors could potentially influence chemical and mechanical properties. Finally, compostable plastics may one day completely replace single-use fossil fuel-based plastics because of their minimal impact on the environment. Understanding the effect of different components and additives opens possibilities for optimizing the design of fully compostable products.

## Figures and Tables

**Table 1:** Film thickness, tensile strength, and elongation at break of CC:U and AA:G TPS films.

TPS Film Sample	Film Thickness (mm)	Tensile Strength (MPa)	Elongation at Break (%)
CC:U	0.180 (SD = 0.025)	0.176 (SD = 0.080)	47.16 (SD = 3.57)
AA:G	0.190 (SD = 0.013)	2.037 (SD = 1.240)	31.08 (SD = 12.59)

## Data Availability

Data will be made available upon request.
